# Larval Indices of Vector Mosquitoes as Predictors of Dengue Epidemics: An Approach to Manage Dengue Outbreaks Based on Entomological Parameters in the Districts of Colombo and Kandy, Sri Lanka

**DOI:** 10.1155/2020/6386952

**Published:** 2020-06-16

**Authors:** Lahiru Udayanga, Subashinie Aryaprema, Nayana Gunathilaka, M. C. M. Iqbal, Thilan Fernando, W. Abeyewickreme

**Affiliations:** ^1^Faculty of Medicine, University of Kelaniya, Ragama, Sri Lanka; ^2^Department of Biosystems Engineering, Faculty of Agriculture & Plantation Management, Wayamba University of Sri Lanka, Makadura, Sri Lanka; ^3^Regional Entomologist-Western Province, Colombo, Sri Lanka; ^4^Plant and Environmental Sciences, National Institute of Fundamental Studies, Kandy, Sri Lanka; ^5^Anti Malaria Campaign, Ministry of Health, Colombo 5, Sri Lanka; ^6^Department of Para clinical Sciences, Faculty of Medicine, Sir John Kotelawala Defense University, Rathmalana, Sri Lanka

## Abstract

**Background:**

Early detection of dengue epidemics is a vital aspect in control programmes. Predictions based on larval indices of disease vectors are widely used in dengue control, with defined threshold values. However, there is no set threshold in Sri Lanka at the national or regional levels for *Aedes* larval indices. Therefore, the current study aimed at developing threshold values for vector indices in two dengue high-risk districts in Sri Lanka.

**Methods:**

Monthly vector indices (House Index [HI], Container Index [CI], Breteau Index for *Aedes aegypti* [BI_agp_], and *Ae. albopictus* [BI_alb_]), of ten selected dengue high-risk Medical Officer of Health (MOH) areas located in Colombo and Kandy districts, were collected from January 2010 to June 2019, along with monthly reported dengue cases. Receiver Operating Characteristic (ROC) curve analysis in SPSS (version 23) was used to assess the discriminative power of the larval indices in identifying dengue epidemics and to develop thresholds for the dengue epidemic management.

**Results:**

Only HI and BI_agp_ denoted significant associations with dengue epidemics at lag periods of one and two months. Based on *Ae. aegypti*, average threshold values were defined for Colombo as Low Risk (2.4 ≤ BI_agp_ < 3.8), Moderate Risk (3.8 ≤ BI_agp_ < 5), High Risk (BI_agp_ ≥ 5), along with BI_agp_ 2.9 ≤ BI_agp_ < 4.2 (Low Risk), 4.2 ≤ BI_agp_ < 5.3 (Moderate Risk), and BI_agp_ ≥ 5.3 (High Risk) for Kandy. Further, 5.5 ≤ HI < 8.9, 8.9 ≤ HI < 11.9, and HI ≥ 11.9 were defined as Low Risk, Moderate Risk, and High Risk average thresholds for HI in Colombo, while 6.9 ≤ HI < 9.1 (Low Risk), 8.9 ≥ HI < 11.8 (Moderate Risk), and HI ≥ 11.8 (High Risk) were defined for Kandy.

**Conclusions:**

The defined threshold values for *Ae. aegypti* and HI could be recommended as indicators for early detection of dengue epidemics and to drive vector management activities, with the objective of managing dengue epidemics with optimal usage of financial, technical, and human resources in Sri Lanka.

## 1. Background

*Aedes aegypti* and *Ae. albopictus* are considered as efficient vectors for transmission of arboviral diseases such as dengue, chikungunya, and zika [1]. At the global level, dengue has become a public health importance in more than 128 countries recording approximately 390 million cases annually, with 3.9 billion people at the risk for dengue [2–3]. Significant progression in urbanization, transport, and geographical expansion of dengue vectors have contributed further to elevate the disease burden, resulting in adverse impacts on economic development in many countries, due to losing of productivity and higher expenditure associated with control efforts [4–6]. Although the first record of dengue was identified in the mid-1960's in Sri Lanka [7], the actual threat as a public health importance resulted gradually after 1989. The worst ever epidemic of dengue was witnessed in 2017 with 186,101 cases, along with more than 390 deaths. In the year 2019, a total of 105,049 cases were recorded, which was second only to the epidemic caused in 2017 [8].

*Ae. aegypti* and *Ae. albopictus* female adults primarily depend upon humans to cater for their dietary needs, causing them to coevolve with humans. This has enabled them to occupy different environments (urban, semiurban, and rural) and oviposit in a variety of natural (leaf axils, small ponds, and tree holes, etc.) and artificial (such as discarded containers, tires, gutters, and cement tanks, etc.) habitats [9–11]. Also, they are capable of withstanding phenomenal climatic stress factors with different morphological and behavioural adaptations such as desiccation resistant eggs, skipping oviposition, multiple blood feeding, and dormancy ability of eggs [12, 13]. Further, in recent years, the *Aedes* vectors have adapted to oviposit in unusual breeding habitats such as in saline and organically polluted water [12]. Therefore, *Aedes* vectors have become smart survivors in today's dynamic environment.

Due to the unavailability of a successful prophylactic or curative treatment thus far until now, vector control is the main driving force in dengue control programmes [14–15]. Vector control programmes consist of a variety of approaches including mechanical/physical, chemical, and biological efforts to reduce vector density and vulnerability [16]. Similar to many developing countries, Vector Controlling Entities (VCE) in Sri Lanka have already implemented Integrated Vector Management (IVM) programmes with the intention of maintaining vector densities at lower levels based on entomological indices.

Regardless of different entomological indices that capture various life stages of vector mosquitoes such as larvae [18–20], pupae [21–22], and adults [18, 23], Sri Lankan VCE still rely upon *Aedes* larval indices that focus on the immature stages of dengue vectors. *Aedes* larval indices (House Index [HI], Breteau Index [BI], and Container Index [CI]) are widely used in many developing countries, due to their convenient applicability, and restrictions in human and financial resources [17]. However, the reliability and sensitivity of larval indices and threshold values are now being questioned by several studies [21, 24]. Therefore, the use of *Aedes* larval indices with well-defined threshold values, considering the microlevel dynamics of dengue vectors, can result in successful entomological management of dengue, through the provision of reliable prealerts on probable epidemics [25–27]. Therefore, the development of reliable threshold values for the entomological management of dengue vectors is essential to control dengue in developing countries like Sri Lanka.

The current vector control activities within Sri Lanka are mainly driven based on BI and HI [17]. Chemical fogging, the routine vector reduction strategy, is practiced when the BI value exceeds 5%, with reported dengue cases in a considering area or when BI > 20% even without reported cases. All the other scenarios are usually recommended to be dealt with source reduction and elimination of breeding sites [17, 28]. However, changes in the environmental factors (microclimatic variations, natural vegetation cover, and land-use), characteristics of the local communities (sociocultural practices, herd immunity variations, and migratory patterns) and vector populations (presence of the dengue virus and stereotypic variations), may significantly affect the local transmission of dengue and incidence of dengue epidemics [17, 19, 20, 25]. Therefore, defining thresholds for local situations is more sensible and appropriate for dengue vector management in control programmes [17].

Setting up a reliable threshold has become a challenging task, due to few major knowledge gaps such as defining an outbreak of dengue, dengue risk situations (in terms of cases), type of mosquito density required to reflect the risk, method to correlate the epidemiological, and entomological cases and spatial scale to be considered [17, 29]. Some efforts based on Receiver Operating Characteristic curves (ROC), geoinformatics based approaches, mathematical models, and frequency analysis of vector abundance, etc. have been used by previous researchers for defining critical values for dengue epidemic management in other countries [17, 30–31]. However, Sri Lankan VCE still rely upon the previously mentioned threshold for BI in dengue epidemic management, which may have been derived from thresholds used for yellow fever in 1920 [17, 21]. Therefore, the current study intends to propose threshold values to facilitate dengue epidemic management, based on entomological and epidemiological data by using Receiver Operating Characteristic curves (ROC) in two selected dengue high-risk urban areas in Sri Lanka.

## 2. Method

### 2.1. Study Area

**Figure 1:**
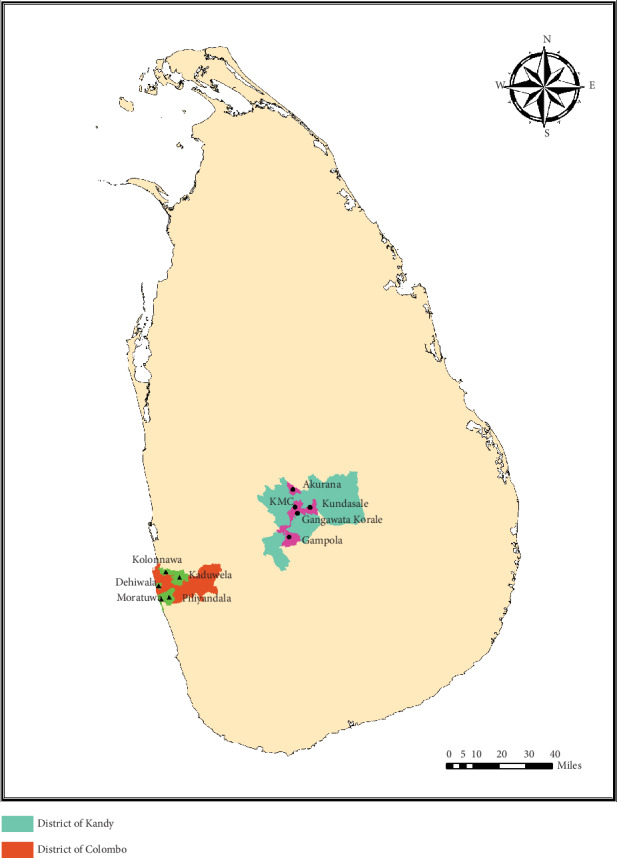
Location of the studied MOH areas in the districts of Colombo (Dehiwala, Kaduwela, Kolonnawa, Moratuwa, and Piliyandala) and Kandy (Akurana, Gampola, Gangawata Korale, Kandy Municipal Council, and Kundasale) within Sri Lanka.

The study was conducted in two major districts of Sri Lanka; Colombo (6.70° to 6.98°N and 79.83° to 80.22°E) and Kandy (6.93° to 7.50°N and 80.43° to 81.04°E) as indicated in Figure 1. Colombo district has an area of 699 km^2^ with 2,309,809 habitants and is the commercial capital of Sri Lanka [32]. It is divided into 16 Medical Officers of Health (MOH) areas established for the management of health concerns at the regional level. The district of Kandy covers an area of 1,940 km^2^ with a population of 1,369,899 and consists of 23 MOH areas [33]. Colombo and Kandy districts were the first (*n* = 20, 718 dengue cases) and third (*n* = 8, 940 dengue cases) high-risk districts of the country in 2019, respectively. Five MOH areas, that reported the highest number of dengue cases during the period of 2013–2019, were selected from each district.

### 2.2. Data Collection

Routine entomological surveillance activities were conducted on a monthly basis within the selected eight MOH areas from January 2016, to June 2019, by following standard dipping, siphoning, and pipetting methods as recommended by the National Dengue Control Unit, Sri Lanka [34]. The larval surveillance activities were conducted at the household level after acquiring the informed written consent from the household heads allowing to conduct the entomological surveillance during the study period. The collected specimens were identified up to the species level by trained entomological staff based on the standard morphological keys [35].

Meanwhile, past entomological surveillance data corresponding to the period of January, 2010, to June 2019, were also collected at the monthly scale from the MOH offices along with the number of reported dengue cases covering the entire study period (January 2010 to June 2019).

### 2.3. Data Management and Statistical Analysis

House Index [HI] (percentage of houses positive for *Aedes* larvae) and Breteau Index [BI] (number of positive containers with *Aedes* larvae per 100 houses) and Container Index [CI] (number of positive containers with *Aedes* larvae per 100 containers) were calculated in accordance with the WHO guidelines [36]. Pearson's correlation analysis was used to identify the association between the larval indices and the number of reported dengue cases at MOH level at different lag periods. Larval indices that denoted a significant association (*P* < 0.05 at 95%) were considered as the discriminative indices to define dengue epidemics.

Receiver Operating Characteristic (ROC) curves were used to assess the discriminative power of three larval indices (namely, House Index [HI], Breteau Index for *Ae. aegypti* [BI_agp_] and *Ae. albopictus* [BI_alb_], and Container Index [CI]) to identifying dengue epidemics. The area under the curve (AUC) measures the total two-dimensional area that lies under the ROC curve. It was used to classify larval indices into three groups: (i) Noninformative (AUC ≤ 0.5), (ii) Moderately accurate (0.5 < AUC ≤ 0.7), and (iii) Highly accurate (0.7 < AUC ≤ 1), by modifying the recommendations of Luo et al. [27] and Sanchez et al. [25]. Exceedance of the monthly cases by mean plus two standard deviations of the preceding five years was used as the definition of dengue epidemics during the analysis by modifying the recommendations of Badurdeen et al. [37] due to the shortage of a widely accepted definition for dengue outbreaks.

The location of the maximum vertical distance from the line of equality (reference line) or in other words the points on the ROC curve located farthest from the line of equality was selected as threshold values based on the Youden index, to discriminate different phases in the dengue incidence [38]. In this case, three major points were selected as preepidemic (low risk), epidemic (risk), and high-risk phases. The optimal threshold points for dengue epidemic management were established for each larval index in all selected MOH areas. The SPSS (version 23) was used for all statistical analyses.

### 2.4. Ethical Aspects

Ethical approval was obtained from the Ethics Review Committee of the Faculty of Medicine, University of Kelaniya (P/155/10/2015).

## 3. Results

### 3.1. Seasonality and Distribution of Vector Indices

**Figure 2:**
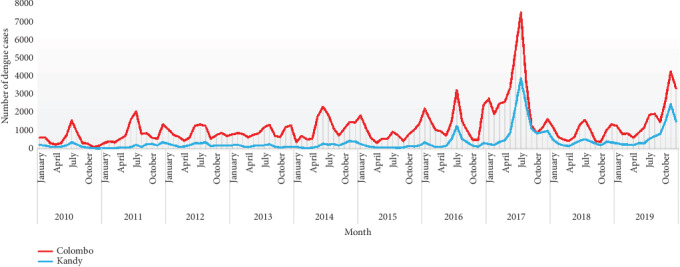
Temporal variation of reported dengue cases in Colombo and Kandy districts of Sri Lanka.

The temporal variations of average annual vector indices, with respect to BI values of both *Ae. aegypti* (BI_agp_) and *Ae. albopictus* (BI_alb_), HI, and CI from January 2010 to June 2019 are illustrated in S1 appendix. Seasonal fluctuation pattern of larval indices was observed in the districts of Colombo and Kandy (Figure 2; Supplementary Figures S1–S4). The temporal variation of reported dengue cases reflected two major epidemic cycles; (i) April to July and (ii) October to December/January, annually with the epidemic peak in June-July and December-January, respectively, in each cycle (Figure 2). However, the highest number of cases has been reported during the epidemic cycle in April to July.

### 3.2. Association between Larval Indices and Occurrence of Dengue Epidemics

According to the Pearson correlation analysis, *Ae. aegypti* (BI_agp_) had the significantly strongest association with dengue cases reported in all MOH areas after lag periods of one (0.387 ≥ *r* ≤ 0.580) and two (0.587 ≥ *r* ≤ 0.767) months (*P* < 0.05). On the other hand, HI had a significantly negative association with dengue cases after one- and two-month lag periods (Table 1). The highest correlation coefficients of BI_agp_ and HI were observed after two months lag period. Regardless of the positive correlation between CI and dengue cases, the relationship was nonsignificant and often poor (*r* < 0.330), except in Akurana (*r* = 0.385). In summary, only BI_agp_ and HI denoted significant associations with the incidence of dengue cases, and therefore, the above two indices could be used as important parameters for epidemic management via vector control approaches.

### 3.3. Discriminative Indicators of Dengue Epidemics according to ROC

Since the lag periods of one and two months resulted in significant associations, the ROC was also conducted for one and two month lag periods. At one-month lag period, only BI_agp_ of Akurana (0.530) and Moratuwa (0.507) had moderately accurate discriminative powers in reflecting dengue epidemics as shown in Table 2. With 2 months lag period, BI_agp_ of Moratuwa (0.794), Akurana (0.761), and Kaduwela (0.731) denoted highly accurate discrimination of dengue epidemics, while moderate discriminative power was shown by BI_agp_ in all the other study areas (Table 2). On the other hand, HI denoted moderately accurate discrimination of dengue epidemics in all the MOH areas, while BI_alb_ and CI indicated noninformative reflections of dengue epidemics. Therefore, based on the AUC values, only BI_agp_ and HI could be recognized as larval indices, which are suitable for the prediction of dengue epidemics in all MOH areas at two months lag period.

### 3.4. Threshold Values

**Figure 3:**
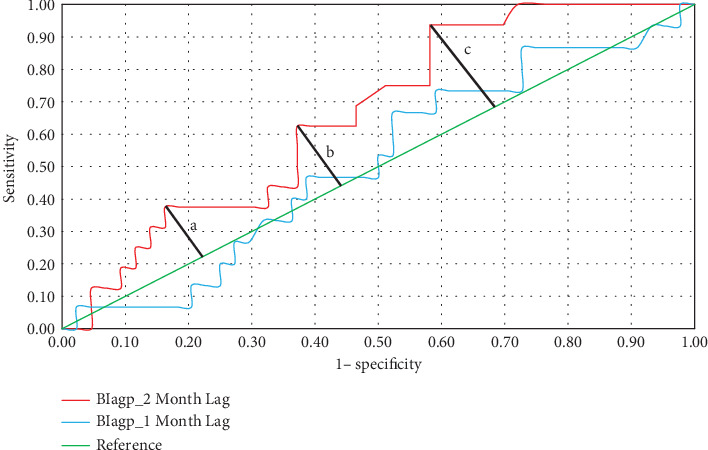
Receiver Operating Characteristic curve derived for the Akurana MOH area along with maximum vertical distance from line of equality corresponding to (a) Low risk; (b) Moderate risk; and (c) High-risk thresholds of BI_agp_ at a lag period of two months.

Among the considered larval indices, only BI_agp_ and HI emerged as discriminative indicators of dengue epidemics. Therefore, three threshold levels were defined for these as “Low Risk”, “Moderate Risk”, and “High Risk” phases, to facilitate management of dengue vectors based on the association of *Aedes* vectors with dengue incidence (Table 3). A larval index value lower than the “Low Risk” threshold indicates a preepidemic phase, where there is no risk of dengue occurrence. Exceeding the “Low Risk” and “Moderate Risk” thresholds indicates the low risk and moderate risk phases of a dengue epidemic, respectively. Finally, the High-risk phase of an epidemic is denoted when the “High Risk” threshold is exceeded by the relevant larval index value. The delineation of the risk thresholds for BI_agp_ in Akurana MOH by using the ROC curve and Youden index is depicted in Figure 3, as an example.

The minimum “Low Risk”, “Moderate Risk”, and “High Risk” threshold values for BI_agp_ were recommended for Kolonnawa MOH area as 2.0, 3.5, and 4.4, respectively. On the other hand, the maximum threshold values were set for the Gampola MOH area as 3.9, 5.1, and 6.2 (“Low”, “Moderate”, and “High Risk” thresholds), respectively. In the case of HI, the minimum “Low Risk” cutoff value (4.7) was suggested for Piliyandala MOH area (located in the district of Colombo), while the minimum “Moderate Risk” (7.7) and “High Risk” thresholds were identified for Akurana MOH in Kandy. The Piliyandala MOH area had the maximum “High Risk” threshold of 16.3 for HI, while Gampola had the maximum “Low Risk (7.8)” and “Moderate Risk (12.1)” thresholds (Table 3).

## 4. Discussion

Comparison of entomological indices that reflect the incidence of dengue epidemics and the establishment of reflective thresholds has remained a challenge to many dengue-endemic countries, including Sri Lanka [17, 24]. However, few studies have emphasized the capability of different vector indices for quantitative reflection of dengue outbreaks [24], while several other studies have queried the competency of defined vector indices-based thresholds in managing dengue epidemics through predictions [17, 25, 39].

A diverse interplay of multiple factors such as humans, *Aedes* vectors, dengue virus strains, and environmental considerations (climate, land use, vegetation, and degree of urbanization, etc.) directly influences the disease incidence and nature of dengue epidemics [19, 20, 40–42]. Thresholds for larval indices should be defined at regional levels, encompassing other factors such as population dynamics of vectors at microlevel, herd immunity, human population characteristics, surrounding environment, and existing vector management practices [17]. Consideration of administrative boundaries, negligence, or limited focus on serotype prevalence of dengue virus, difficulties in tracking the herd immunity, and human mobility could be recognized as major limitations that may directly influence poor reflection of dengue epidemics via early warning systems [19, 20, 24].

Even though the density of *Ae. albopictus* was relatively higher in all study areas, a nonsignificant association was indicated by BI_alb_ during both correlation and ROC analysis. Recent studies conducted in Sri Lanka have also evidenced high prevalence rates of *Ae. albopictus* reflected by the BI_alb_, which has been concluded as the vector that maintains the dengue viral strains, but with a lesser contribution towards transmission to humans [17, 43, 44]. However, *Ae. albopictus* was abundant even in the urban and semiurban areas such as Kolonnawa, Kandy Municipal Council (KMC), Piliyandala, and Gampola. The presence of adequate vegetation and potential breeding sites could be the leading factors for the occurrence of *Ae. albopictus* [17, 43]. On the other hand, *Ae. aegypti* indicated a positive moderate to strong association with the reported dengue cases in all MOH areas after a lag period of two months. Therefore, *Ae. aegypti* could be recognized as the major transmitting agent of dengue within the studied MOH areas, as depicted by the higher association of BI_agp_ with the epidemics of dengue [43, 44]. Therefore, BI_agp_ could be recommended as the key larval index that reflects the incidence of dengue epidemics.

Irrespective of using both CI and HI in routine entomological surveillance, only HI stated a significant and moderate relationship with dengue cases. In general, the HI is influenced by densities of both *Ae. albopictus* and *Ae. aegypti.* A notable negative association has been observed among the vector densities of *Ae. albopictus* and *Ae. aegypti* in the study areas. This is evidenced by the decrease of BI_alb_, while BI_agp_ increased prior to and within dengue epidemics [17], which indirectly triggered HI to decline during dengue epidemic periods, as density of *Ae. albopictus* declines. This may be the reason for the negative association among the HI and dengue outbreak incidence within the studied MOH areas. The efficacy of CI is questionable due to some limitations such as incompetency to reflect the number of positive containers per house, per area, per person, and qualitative nature, implying a nondiscriminative power in defining dengue epidemics [17]. Therefore, in the present study, only BI_agp_ and HI were recognized as reflective indices to predict dengue epidemics in the investigated study areas. The findings of the present study are also in agreement with previous investigations conducted in Sri Lanka [17, 44, 45].

Thresholds developed based on BI and HI have been successfully used in dengue epidemic prediction and management in Brazil, Havana, Thailand, Trinidad, and America [40, 41, 45–47]. The current study has derived three risk thresholds for BI_agp_ and HI individually at the regional level (MOH level). For Colombo district, 2.4 ≤ BI_agp_ ≤ 3.7, 3.8 ≤ BI_agp_ < 5, and BI_agp_ ≥ 5 were defined as the average BI_agp_ based risk thresholds depicting the “Low”, Moderate”, and “High Risk” phases of dengue epidemics, respectively. In the district of Kandy, the average BI_agp_ based risk thresholds was relatively higher as 3.0 ≤ BI_agp_ ≤ 4.1 (Low Risk), 4.2 ≤ BI_agp_ < 5.3 (Moderate Risk), and BI_agp_ ≥ 5.3 (High Risk). In the case of HI, the average thresholds for Colombo district were defined as Low Risk (5.5 ≤ HI ≤ 8.8), Moderate Risk (8.9 ≥ HI < 11.9), and High Risk (HI ≥ 11.9), while 6.9 ≤ HI ≤ 9.0 (Low Risk), 9.1 ≤ HI < 11.8 (Moderate Risk), and HI ≥ 11.8 (High Risk) are recommended for Kandy. These average thresholds are proposed to be applied in other MOH areas located in the district, while the MOH areas investigated in this study could rely upon specific thresholds developed in the current study.

In a recent study, Ong et al. [48] have used the Breeding Percent (BP) as an entomological index, which is the proportion of *Ae. aegypti* positive breeding sites to the total number of *Aedes* species positive breeding sites (*Ae aegypti* and *Ae. albopictus*) in Singapore, to investigate the risk of geographical expansion of *Ae aegypti*. Their findings emphasize that areas with a higher *Ae. aegypti* BP have a higher degree of geographical expansion of *Ae aegypti*. Further, such areas tend to have higher case numbers suggesting that the BP of *Ae. aegypti* is a key factor for the risk characterization of dengue fever in endemic countries, like Sri Lanka [48]. A similar efficacy was noted with the use of BI_agp_ that directly reflects larval abundance of *Ae. aegypti* in the current study.

In a previous study, a set of entomologically driven thresholds have been developed for a selected set of MOH areas in Kandy, solely based on the natural dynamics of dengue vectors [17]. The current study has further advanced by incorporating epidemiological data (reported dengue cases) with the entomological data, to derive more reliable and reflective thresholds for dengue epidemic management. The thresholds defined for BI_agp_ and HI in Akurana, KMC & GK, and Gampola MOH areas (in the abovesaid previous study) are in close agreement to the threshold values derived by the current ROC approach [17].

As a routine practice, the VCE in Sri Lanka utilize BI > 20 (without dengue cases) or BI > 5 (with dengue cases) as the risk threshold for guiding vector control activities. However, these risk levels are often insensitive to the local variations in epidemiological and entomological aspects. Further, even though cumulative BI (that accounts for both *Ae. aegypti* and *Ae. albopictus*) could exceed 5 (with cases) or 20 (without cases), it could overlook the actual risk for epidemic incidence as *Ae. albopictus*, which is abundant in the environment, could heavily influence the BI [17, 28].

Thresholds defined for BI_agp_ and HI denoted a notable degree of spatial variation among the studied MOH areas. As mentioned previously, vector densities are one of the contributing risk factors of dengue incidence, in addition to climatic conditions, land use, population density, degree of herd immunity, etc. [41, 49, 50]. Thus, a complex interplay of such factors influences the incidence and severity of dengue epidemics in a considering area [17, 51]. Interestingly, the *Aedes* vector density is clearly influenced by most of these factors such as meteorological parameters, land use, meteorological factors, and practices among the local communities, etc. [17, 48, 52]. The contrasting variations in environmental conditions (both meteorological and land use) practices among the local communities and degree of urbanization among the studied MOH areas could be the reason behind the spatial discriminations in both vector densities and thereby threshold levels [17, 52–54].

### 4.1. Recommended Interventions for Risk Phases

The current vector controlling approaches heavily rely upon chemical-based methods (systematic use of larvicides, adulticides, and chemical fogging), along with the physical elimination of breeding sites of *Aedes* vectors. Furthermore, exceeding the BI threshold is dealt with intensive chemical fogging within the relevant locality [17]. Through the current approach of thresholds and recommended actions, an attempt is made to utilize the principles of IVM such as community mobilization, awareness raising, and capacity building, in addition to the chemical-based vector control. The suggested approach for dengue epidemic management, which is derived based on entomological and epidemiological aspects, would be more ecofriendly and community responsive. Further, it would also facilitate the current national concerns to become environmentally friendly and green. Continuous entomological surveillance activities and evaluation strategies are also recommended to be implemented to investigate the efficacy of the proposed thresholds and interventions for dengue epidemic management.

The absence of records on serological circulations of dengue viruses, limitations in the availability of long term surveillance data (arising due to limitations in knowledge, trained staff, financial and physical resources, limited commitment and satisfaction of field staff) and limited knowledge on herd immunity, is the major limitations encountered in defining the thresholds in the current study [17]. Further, monthly records of epidemiological and entomological data were used in the current study due to limitations in some data availability. The use of weekly entomological data could have yielded more reliable associations among larval indices and dengue cases, which could facilitate the development of better thresholds than from monthly data. However, consideration of entomological and epidemiological aspects at the local level may compensate for the aforesaid limitations [17].

In Sri Lanka, VCE are struggling to manage dengue epidemics through a variety of approaches such as vector control, environmental, and patient management [34]. However, containing dengue epidemics has remained a challenge, mainly due to limited resources and difficulties in taking early responsive actions [17]. Previous studies have clearly shown that the emergency control measures such as the use of chemical insecticides are less effective in controlling dengue, once cases begin to get reported [55]. Therefore, the thresholds defined under this study for BI_agp_ plays a critical role in such circumstances as an early warning signal two-months ahead (two-month lag), providing a valuable window of opportunity for the VCEs to design and initiate vector control activities, before the incidence of a dengue epidemic. This will be a critical determinant in the dengue epidemic management as it can ensure the timely containment of *Aedes* vectors prior to an outbreak incidence [18, 25, 48]. Even though several studies have suggested that use of adult female surveillance indices would be more accurate to guide the vector control activities [21], this may not provide an adequate time for disease management since it provides only a smaller lag period to suppress vector population, prior to transmission of the virus to the human population [24]. Therefore, the threshold values of BI_agp_ and HI recommended in this study with two months lag period would be of immense importance in responding to dengue epidemics prior to incidence [55].

In light of this, thresholds defined in this study would be helpful for adopting and directing vector control activities, with optimum utilization of limited human and financial resources to control dengue epidemics [48, 52]. Therefore, VCE in relevant study areas are recommended to apply the above-developed thresholds in epidemic prediction and management, while evaluating the practical feasibility of the thresholds, subjecting to further modifications and adjustments.

## 5. Conclusion

Based on our findings, both HI and BI_agp_ are significantly associated with dengue epidemics at lag periods of one and two months, emerging as representative *stegomyia* indices capable of defining dengue epidemics. The BI_agp_ ≤ 2.4 (Low Risk), 3.8 ≤ BI_agp_ < 5 (Moderate Risk), and BI_agp_ ≥ 5 (High Risk) are recommended as the average BI_agp_ based risk thresholds for Colombo, while BI_agp_ ≤ 3.0 (Low Risk), 4.2 ≤ BI_agp_ < 5.3 (Moderate Risk), and BI_agp_ ≥ 5.3 (High Risk) are suggested for Kandy. The average thresholds for HI in Colombo are HI ≤ 5.5 (Low Risk), 8.9 ≥ HI < 11.9 (Moderate Risk), and for Kandy are HI ≤ 6.9 (Low Risk), 9.1 ≤ HI < 11.8 (Moderate Risk), and HI ≥ 11.8 (High Risk). The thresholds derived from this study are recommended to be practiced and validated within the studied areas to suppress dengue vectors before an epidemic, through optimum usage of limited financial and human resources. Furthermore, VCE are recommended to conduct properly planned routine vector surveillance activities to assist the dengue epidemic management based on these thresholds. The present study warrants the essential need in developing entomological thresholds in other areas in the country and use of such prediction approaches in dengue control programmes in Sri Lanka.

## Figures and Tables

**Table 1:** Pearson's correlation coefficients for the association between the dengue cases and the larval indices at lag periods of zero, one, and two months in the study areas.

District	MOH area	Lag = 0 month				Lag = 1 month				Lag = 2 months			
		BI_agp_	BI_alb_	HI	CI	BI_agp_	BI_alb_	HI	CI	BI_agp_	BI_alb_	HI	CI
Colombo	Dehiwala	0.309	-0.181	-0.119	0.120	0.471^∗^	-0.270	-0.287	0.197	0.685^∗^	-0.287	-0.387^∗^	0.158
	Kaduwela	0.357	-0.212	-0.149	0.046	0.479^∗^	-0.294	-0.304^∗^	0.102	0.742^∗^	-0.321	-0.574^∗^	0.194
	Kolonnawa	0.337	-0.146	-0.209	0.141	0.439^∗^	-0.242	-0.357^∗^	0.189	0.724^∗^	-0.287	-0.491^∗^	0.234
	Piliyandala	0.243	-0.241	-0.278	0.059	0.411^∗^	-0.267	-0.378^∗^	0.146	0.587^∗^	-0.367	-0.472^∗^	0.159
	Moratuwa	0.342^∗^	-0.175	-0.267	0.173	0.580^∗^	0.294	-0.352^∗^	0.214	0.767^∗^	-0.285	-0.549^∗^	0.167

Kandy	KMC & GK	0.321	-0.254	-0.278	0.148	0.459^∗^	-0.347	-0.480^∗^	0.281	0.764^∗^	-0.310	-0.512^∗^	0.375
	Akurana	0.313	-0.310	-0.307	0.211	0.419^∗^	-0.358	-0.368^∗^	0.385	0.676^∗^	-0.362	-0.387^∗^	0.349
	Gampola	0.287	-0.291	-0.235	0.165	0.387	-0.371	-0.379^∗^	0.293	0.648^∗^	-0.354	-0.413^∗^	0.194
	Kundasale	0.257	-0.243	-0.269	0.107	0.409^∗^	-0.310	-0.374	0.189	0.629^∗^	-0.297	0.398^∗^	0.204

Note: “^∗^” indicate significant relationships among the dengue cases and larval indices at different lag periods, where *P* < 0.05 at 95% level of confidence.

**Table 2:** Area under the Receiver Operating Characteristic curves of different larval indices in terms of dengue epidemic incidence at lag periods of 1 and 2 months.

District	MOH area	Lag = 1 month				Lag = 2 months			
		BI_agp_	BI_alb_	CI	HI	BI_agp_	BI_alb_	CI	HI
Colombo	Dehiwala	0.409	0.289	0.210	0.293	0.661^∗^	0.347	0.319	0.508^∗^
	Kaduwela	0.437	0.319	0.196	0.237	0.731^∗^	0.374	0.287	0.407
	Kolonnawa	0.497	0.217	0.176	0.336	0.698^∗^	0.288	0.269	0.568^∗^
	Piliyandala	0.373	0.327	0.133	0.299	0.619^∗^	0.365	0.301	0.509^∗^
	Moratuwa	0.507^∗^	0.304	0.219	0.361	0.794^∗^	0.354	0.334	0.589^∗^

Kandy	KMC & GK	0.485	0.327	0.297	0.402	0.684^∗^	0.349	0.358	0.519^∗^
	Akurana	0.530^∗^	0.315	0.250	0.456	0.761^∗^	0.317	0.336	0.637^∗^
	Gampola	0.421	0.276	0.184	0.393	0.627^∗^	0.321	0.317	0.573^∗^
	Kundasale	0.389	0.269	0.201	0.243	0.655^∗^	0.309	0.309	0.549^∗^

Note: “^∗^” indicate instances, where area under curve >0.5.

**Table 3:** Recommended risk threshold values for BI_agp_ and HI based on the Receiver Operating Characteristic curves (ROC).

District	MOH area	BI_agp_			HI		
		Low risk	Moderate risk	High risk	Low risk	Moderate risk	High risk
Colombo	Dehiwala	2.1	3.9	4.9	5.8	7.9	9.6
	Kaduwela	2.4	3.7	5.3	5.6	9.2	11.2
	Kolonnawa	2.0	3.5	4.4	5.9	8.3	12.2
	Piliyandala	3.2	4.8	5.7	4.7	11.4	16.3
	Moratuwa	2.1	3.3	4.8	5.5	7.8	10.2
	Average	2.4	3.8	5.0	5.5	8.9	11.9

Kandy	KMC & GK	2.2	3.5	4.7	6.6	8.5	10.6
	Akurana	2.1	3.9	4.8	6.2	7.7	9.9
	Gampola	3.9	5.1	5.9	7.8	12.1	15.5
	Kundasale	3.6	4.1	5.6	7.1	8.1	11.1
	Average	2.9	4.2	5.3	6.9	9.1	11.8

Note: “_∗_” indicate instances, where area under curve >0.5.

## Data Availability

The datasets supporting the conclusions of this article are included within the article and its additional files.

## Abbreviations

BI: Breteau index
BI_agp_: Breteau index for *Ae. aegypti*
BI_alb_: Breteau index for *Ae. albopictus*
CI: Container index
GK: Gangawata Korale
HI: House index
IVM: Integrated vector management
KMC: Kandy municipal council
MOH: Medical officer of health
r: Pearson correlation coefficients
ROC: Receiver operating characteristic curve
VCE: Vector controlling entities.

## Supplementary Materials

Supplementary Materials Supplementary Figures S1–S4: temporal variations in the average Aedes larval indices for each MOH area in the districts of Colombo and Kandy (2010 to 2019).
