# Supplementary material for: Larval Indices of Vector Mosquitoes as Predictors of Dengue Epidemics: An Approach to Manage Dengue Outbreaks Based on Entomological Parameters in the Districts of Colombo and Kandy, Sri Lanka
Source: Biomed Res Int. 2020 Jun 16;2020:6386952. doi: 10.1155/2020/6386952 (PMC7317327; doi:10.1155/2020/6386952)
Supplement: Supplementary Materials — Supplementary Figures S1–S4: temporal variations in the average Aedes larval indices for each MOH area in the districts of Colombo and Kandy (2010 to 2019). [file 6386952.f1.docx]

**Figure S1**. Temporal variations in the monthly average Breteau Index for *Aedes aegypti* (BI_agp_) in the studied MOH areas (2010 to 2019)

**Figure S2**. Temporal variations in the monthly average Breteau Index for *Aedes albopictus* (BI_alb_) in the studied MOH areas (2010 to 2019)

**Figure S3**. Temporal variations in the monthly average Container Index (CI) in the studied MOH areas (2010 to 2019)

**Figure S4**. Temporal variations in the monthly average Premise Index (PI) in the studied MOH areas (2010 to 2019)
